# Selenium-Containing (Hetero)Aryl Hybrids as Potential Antileishmanial Drug Candidates: In Vitro Screening against *L. amazonensis*

**DOI:** 10.3390/biomedicines12010213

**Published:** 2024-01-17

**Authors:** Maria Helena Fermiano, Amarith Rodrigues das Neves, Fernanda da Silva, Manuella Salustiano Andrade Barros, Camila Barbosa Vieira, André L. Stein, Tiago Elias Allievi Frizon, Antonio Luiz Braga, Carla Cardozo Pinto de Arruda, Eduardo Benedetti Parisotto, Sumbal Saba, Jamal Rafique, Thalita Bachelli Riul

**Affiliations:** 1 Faculdade de Ciências Farmacêuticas, Alimentos e Nutrição (FACFAN), Universidade Federal do Mato Grosso do Sul (UFMS), Campo Grande 79070-900, MS, Brazilamardasneves@gmail.com (A.R.d.N.);; 2 Instituto de Biociências (INBIO), Universidade Federal do Mato Grosso do Sul (UFMS), Campo Grande 79070-900, MS, Brazil; 3 Instituto de Química (INQUI), Universidade Federal do Mato Grosso do Sul (UFMS), Campo Grande 79074-460, MS, Brazil; manuella.salustiano@ufms.br; 4 LABSO, Instituto de Química (IQ), Universidade Federal de Goiás (UFG), Goiânia 74690-900, GO, Brazilsumbalsaba@ufg.br (S.S.); 5 Departamento de Química, Universidade Federal de Mato Grosso (UFMT), Cuiabá 78060-900, MT, Brazil; 6 Departamento de Energia e Sustentabilidade, Universidade Federal de Santa Catarina (UFSC), Campus Araranguá, Araranguá 88905-120, SC, Brazil; 7 Departamento de Química, Universidade Federal de Santa Catarina (UFSC), Florianópolis 88040-970, SC, Brazil

**Keywords:** leishmaniasis, organoselenide, 2,5-dihydrooxazole, indole, chromone, coumarin, *β*-naphthol, 1,3,4-oxadiazole, imidazo[1,2-*a*]pyridine, imidazo[2,1-*b*]thiazole

## Abstract

Leishmaniasis remains a significant global health concern, with current treatments relying on outdated drugs associated with high toxicity, lengthy administration, elevated costs, and drug resistance. Consequently, the urgent need for safer and more effective therapeutic options in leishmaniasis treatment persists. Previous research has highlighted selenium compounds as promising candidates for innovative leishmaniasis therapy. In light of this, a library of 10 selenium-containing diverse compounds was designed and evaluated in this study. These compounds included selenium-substituted indole, coumarin, chromone, oxadiazole, imidazo[1,2-*a*]pyridine, Imidazo[2,1-*b*]thiazole, and oxazole, among others. These compounds were screened against *Leishmania amazonensis* promastigotes and intracellular amastigotes, and their cytotoxicity was assessed in peritoneal macrophages, NIH/3T3, and J774A.1 cells. Among the tested compounds, **MRK-106** and **MRK-108** displayed the highest potency against *L. amazonensis* promastigotes with reduced cytotoxicity. Notably, MRK-106 and MRK-108 exhibited IC_50_ values of 3.97 µM and 4.23 µM, respectively, and most of the tested compounds showed low cytotoxicity in host cells (CC_50_ > 200 µM). Also, compounds **MRK-107** and **MRK-113** showed activity against intracellular amastigotes (IC50 18.31 and 15.93 µM and SI 12.55 and 10.92, respectively). In conclusion, the identified selenium-containing compounds hold potential structures as antileishmanial drug candidates to be further explored in subsequent studies. These findings represent a significant step toward the development of safer and more effective therapies for leishmaniasis, addressing the pressing need for novel and improved treatments.

## 1. Introduction

Leishmaniases encompass a diverse spectrum of diseases, each presenting a wide array of clinical manifestations. These clinical conditions are attributed to various species of the kinetoplastid parasite *Leishmania,* affecting both humans and other mammals dwelling in tropical and subtropical regions worldwide [1,2]. According to the World Health Organization (WHO), today, more than 1 billion people live in areas endemic to leishmaniasis and are at risk of infection. An estimated 30,000 new cases of *Visceral leishmaniasis* (VL) and more than 1 million new cases of *Cutaneous leishmaniasis* (CL) occur annually, with an ongoing burden of 12 million people harboring active infections [3]. The transmission of this pathology is facilitated by blood-feeding female sandflies, with the parasites undergoing a complex life cycle involving two distinct forms: extracellular flagellated promastigotes within the vector and intracellular non-flagellated amastigotes residing within mononuclear phagocytes in the mammalian host [4].

Historically, pentavalent antimony stood as the primary treatment for leishmaniasis. However, it comes burdened with concerns of cardiotoxicity, cirrhosis, pancreatic toxicity, and the risk of resistance development [5]. Consequently, amphotericin B (including lipid formulations) emerged as a secondary option. The repertoire of drugs repurposed for leishmaniasis treatment includes amphotericin B, miltefosine, paromomycin, and pentamidine [6]. Miltefosine has also found its place in treating both VL and CL, offering the benefits of oral administration, high efficacy, and a short treatment course. Nonetheless, its usage is hampered by teratogenicity and the potential for drug resistance [6,7,8]. Hence, there is an immediate need to discover new therapeutic approaches and drug compounds to combat these life-threatening diseases.

Selenium compounds are gaining remarkable prominence in medicinal chemistry, representing a burgeoning frontier in the search for novel antiprotozoal agents [9,10]. The strategic integration of selenium (Se) atoms into organic frameworks offers a promising avenue for the creation of enhanced, disease-specific compounds. Organoselenium compounds are renowned for their diverse pharmacological properties [9,10,11,12,13,14,15,16,17,18,19,20]. Furthermore, the biocompatibility, minimal toxicity, and chemical versatility of selenium have spurred the development of a diverse range of Se-based pharmaceuticals. Research has demonstrated that the incorporation of selenium atoms into small molecules significantly amplifies their bioactivity [21]. In broad terms, organoselenium compounds exhibit medical applications that encompass cancer treatment, managing infections and inflammation, addressing Alzheimer’s disease and depression, as well as providing antioxidant benefits [21]. Intriguingly, selenium has also exhibited favorable effects in combating parasitic diseases, including but not limited to malaria, African trypanosomiasis, Chagas Disease, and intestinal parasites [22]. This highlights the pivotal role of selenium compounds in the pursuit of effective treatments against this parasitic disease [22,23,24]. Various Se-containing compounds have shown antimicrobial properties. Additionally, studies indicate that Se supplementation can reduce parasite burden and ameliorate symptoms associated with *Leishmania* spp. and other trypanosomiasis [22]. Moreover, recent findings have highlighted the in vitro leishmanicidal potential of newly synthesized compounds featuring selenium within their structures.

Similarly, heterocyclic compounds hold a pivotal role in organic chemistry, primarily due to their ubiquitous presence in pharmaceuticals, natural substances, and various chemicals integral to our daily lives [25,26,27,28,29,30]. These compounds are characterized by the presence of one or more heteroatoms within cyclic structures, with or without aromatic properties. Oxygen, nitrogen, phosphorus, and sulfur rank among the most frequently incorporated heteroatoms in a majority of heterocyclic compounds [31]. Around 80% of major commercially available synthetic drugs contain at least one heterocyclic scaffold with a broad spectrum of pharmacological potential, encompassing applications such as antitumor and anti-inflammatory, and it is especially important for several active compounds against microorganisms [32]. In the past few decades, the quest for safer and more efficacious drugs to combat leishmaniasis has spurred a multitude of research initiatives. Researchers worldwide have dedicated extensive efforts to synthesize a wide array of antileishmanial agents, distinguished by their incorporation of diverse heterocyclic moieties [33]. Among these moieties, thiazoles, pyrazoles, pyrimidines, chromanones, and imidazoles stand out, each offering a unique chemical scaffold that has garnered attention in the development of potential antileishmanial therapeutics. [31,32,33]. Considering the biological importance of heteroarenes and the wide spectrum of therapeutic properties of organoselenium compounds, molecular hybridization of these structures demonstrates promising biological properties.

Thus, in line with our continuous interest in discovering and developing new sustainable, efficient methodologies for biologically relevant organoselenides and their biological evaluation [17,19,34,35,36,37,38], this article aims to thoroughly examine the synthetic approaches used to create novel chemical compounds for combating leishmaniasis. Herein, we report the in vitro investigation of the antileishmanial potential of selenium-substituted (hetero)aryl hybrids (indole, coumarin, chromone, oxadiazole, imidazo[1,2-*a*]pyridine, Imidazo[2,1-*b*]thiazole, and oxazole, among others) seeking to assess their efficacy and potential for treating this parasitic disease, with a focus on understanding the relationships between compound structures and their biological activity. Our goal is to contribute to the broader understanding of innovative drug development for addressing this widespread tropical disease.

## 2. Materials and Methods

### 2.1. Synthesis of Selenium-Substituted (Hetero)Aryl Hybrids

**Figure 1:**
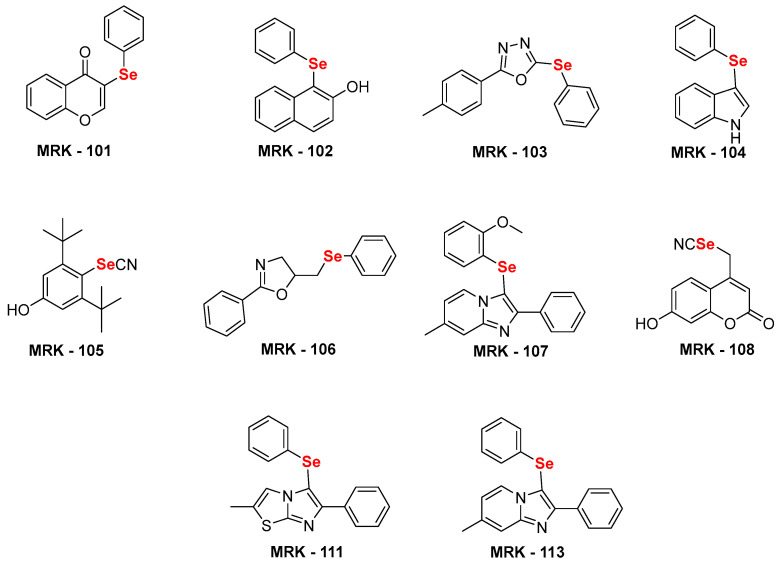
Chemical structure of selenium-substituted (hetero)aryl hybrids [34,35,36,37,38,39,40] used in this study.

For the current study, we selected several nitrogen and oxygen-containing heterocycles. Some privileged naturally occurring heterocycles have also been selected, such as indole, coumarin, and flavanone. In addition, oxadiazole, imidazo[1,2-*a*]pyridine, Imidazo[2,1-*b*]thiazole, and oxazole were also selected. These heterocyclic compounds have been utilized to access structurally diverse selenium-substituted (hetero)aryl hybrids **MRK101–108**, **111**, and **113** (Figure 1) using our previously established sustainable routes. [34,35,36,37,38,39,40].

### 2.2. Mice and Parasites

Female BALB/c mice, aged 6–8 weeks with an average weight of 30 g, were obtained from the Central Animal Facility of the Federal University of Mato Grosso do Sul (UFMS, Campo Grande-MS, Brazil). The animals were housed in individually ventilated cages (IVCs) within a ventilated rack system (Alesco, Monte Mor, Brazil) under specific pathogen-free (SPF) conditions. The environment maintained a temperature of 25 °C ± 1 °C, a 12-h light/dark cycle, and ad libitum access to food (Nuvital, Colombo, Brazil) and water. All procedures were approved by the Ethics Committee on Animal Experiments (CEUA) of UFMS (protocol number 1.041/2019) and adhered to relevant guidelines for animal welfare and research.

*Leishmania amazonensis* (IFLA/BR/1967/PH8 strain) was maintained as promastigotes at 26 °C in Schneider’s medium (Sigma-Aldrich, St. Louis, MO, USA) supplemented with 20% heat-inactivated fetal bovine serum (FBS, Sigma-Aldrich, USA), 10,000 U/mL penicillin, and 10 mg/mL streptomycin (Sigma-Aldrich, USA) for a maximum of 20 serial passages. Parasites in the exponential growth phase were used for the in vitro anti-promastigote assay, while those in the stationary phase were used for the anti-amastigote assay. Intracellular amastigote forms were obtained by infecting murine peritoneal macrophages with promastigotes.

### 2.3. Anti-Promastigotes Assays

Selenium compounds MRK-101 to 113 were evaluated for their anti-promastigote activity against *L. amazonensis* in five replicates. Serial dilutions ranging from 100 to 50, 25, and 12.5 µM were prepared in supplemented Schneider’s Insect Medium (Sigma-Aldrich, USA). The microplates were then seeded with *L. amazonensis* promastigotes (1 × 10^5^ parasites/well) and incubated at 26 °C for 48 h in a Biochemical Oxygen Demand (BOD) incubator (Cienlab, Campinas, Brazil). Cell viability was assessed using the resazurin (Sigma-Aldrich) assay. A solution of resazurin (Sigma-Aldrich, USA) at 0.2 mg·mL^−1^ was added to each well, and after 4 h of incubation at 26 °C, the absorbances at 570 nm and 600 nm were acquired, and the viability calculation for all wells was performed based on the formula and instructions provided by the AlamarBlue^®^ manufacturer’s website (Bio-Rad, Hercules, CA, USA) [41]. Cell viability was evaluated from the optical density and compared to untreated cells. Cells incubated with amphotericin B (1.25, 2.5, 5, and 10 µM) served as the reference anti-promastigote drug, while dimethyl sulfoxide (DMSO, Sigma-Aldrich, USA) in Schneider’s Insect Medium (Sigma-Aldrich, USA) was used as the negative control. The half-maximal inhibitory concentration (IC_50_) values for each compound were calculated using a nonlinear dose–response regression curve generated by Prism 5 (GraphPad Software, USA). To compare variances, ANOVA with Tukey’s post-test was used, supplemented by the parametric *t*-test, with a 95% confidence interval.

### 2.4. Peritoneal Macrophages

Following animal euthanasia, murine peritoneal macrophages were harvested. Ten milliliters of cold RPMI 1640 medium (Sigma-Aldrich, USA) supplemented with 10,000 U/mL penicillin and 10 mg/mL streptomycin (Sigma-Aldrich, USA) was injected into the peritoneal cavity. The abdominal region was gently massaged to facilitate macrophage release. The peritoneal fluid was then aspirated and transferred to an ice-cold Erlenmeyer flask to prevent cell adherence. Cell quantification was performed using a Neubauer’s chamber after trypan blue staining (Sigma-Aldrich, USA) for viability assessment.

### 2.5. Treatment of Infected Macrophages

Murine peritoneal macrophages (1 × 10^6^ cells/well) were seeded in three replicates onto 24-well plates containing round glass coverslips (13 mm) pre-coated with 10% FBS in RPMI 1640 medium (Sigma-Aldrich, USA). Plates were incubated at 37 °C with 5% CO_2_ for 1 h to allow adhesion, which was confirmed by microscopy. Following two washes with Phosphate buffer solution (PBS) (Sigma-Aldrich, USA), adherent macrophages were infected with stationary phase *L. amazonensis* promastigotes (4 × 10^6^ parasites/mL) and incubated at 35 °C/5% CO_2_. After 4 h, free parasites were removed by two PBS washes, and infected cells were treated for 24 h with the compounds at concentrations of 6.25, 12.5, 25, and 50 μM. Amphotericin B (Sigma-Aldrich, USA; 1.25, 2.5, 5, and 10 μM) served as a reference drug, and untreated cells were used as a negative control. Supernatants were removed after treatment, and cells were washed twice with PBS, fixed with Bouin’s solution (Sigma-Aldrich, USA), and stained with Giemsa (Sigma-Aldrich, USA) diluted 1:10 in distilled water. Coverslips were dehydrated through an acetone/xylene gradient (100%, 70%, 50%, 30%, and 100% each from Sigma-Aldrich) and mounted for microscopic visualization. The total number of intracellular amastigotes was counted in 200 cells per coverslip in three replicates using an optical microscope. The half-maximal inhibitory concentration (IC_50_) values were calculated for each compound using a nonlinear regression curve in GraphPad Prism 5.0 software (GraphPad Software, San Diego, CA, USA).

### 2.6. Cytotoxicity Assays

Murine peritoneal macrophages (1 × 10^6^ cells/well), NIH/3T3 cells (ATCC CRL-1658, mouse fibroblasts lineage), and J774A.1 (ATCC, mouse macrophages lineage) were cultured in 96-well plates (1 × 10^5^ cells/well) in RPMI 1640 medium (Sigma-Aldrich, USA) supplemented with 10% FBS (Cultilab, Campinas, Brazil), 10 IU.mL^−1^ of penicillin, and 100 µg·mL^−1^ of streptomycin (Sigma-Aldrich, USA) and adhered overnight at 37 °C and 5% CO_2_. The medium was replaced by fresh RPMI medium with different concentrations of the compounds ranging from 200 to 3.12 µM and incubated for 48 h. Cells treated with amphotericin B (50.0–0.78 µM) served as the antileishmanial drug reference. DMSO (Sigma-Aldrich, USA) at the concentration required to solubilize the highest test sample concentration was used as a control and did not affect cell viability control; fibroblasts cultured with medium alone were used as a life/viability control. Cell viability was assessed using the colorimetric resazurin method [41]. Then, 5 µL of 0.2 mg·mL^−1^ resazurin solution (Sigma-Aldrich, USA) was added to each well and incubated for 4 h at 37 °C and 5% CO_2_. Absorbances were acquired at 570 and 600 nm, and the viability calculation was performed as described in Section 2.3. The half-maximal cytotoxic concentration (CC_50_) for each compound was calculated from a sigmoidal regression of the dose–response curve generated using GraphPad Prism 5.0 software (GraphPad Software, USA). In order to compare variances, ANOVA with Tukey´s post-test was used, supplemented by the parametric *t*-test, with a 95% confidence interval.

## 3. Results

### 3.1. Anti-Promastigotes and Anti-Amastigotes Assays

**Figure 2:**
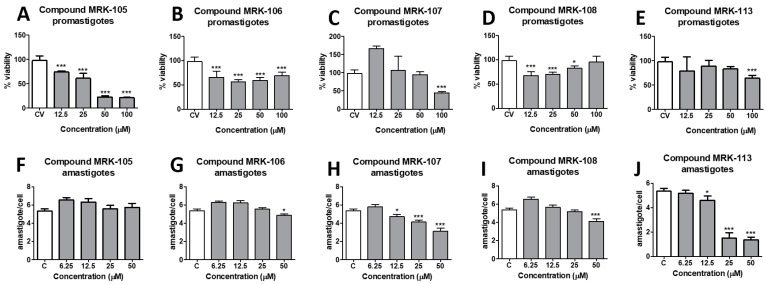
Antileishmanial activity of synthesized selenium compounds **MRK-105**, **MRK-106**, **MRK-107**, **MRK-108**, and **MRK-113** against promastigotes (**A**–**E**, upper graphs) and intracellular amastigotes (**F**–**J**, lower graphs) of *L. amazonensis*. * and *** mean *p* < 0.01 and *p* < 0.001, respectively (ANOVA and Tukey’s post-test).

The antileishmanial activity of the synthesized selenium compounds was evaluated by culturing promastigote forms of *L. amazonensis* in the presence of different concentrations of each molecule. Control wells containing only the culture medium (C) and amphotericin B (ANFB), a reference drug for antileishmanial activity, were also prepared. After 48 h of incubation, the percentage of promastigote form viability was assessed by the resazurin colorimetric method and calculated compared to the control wells. The antileishmanial activity of the selenium compounds was also assessed in intracellular amastigote forms inside infected murine peritoneal macrophages. The representative results obtained for five of the ten compounds tested are illustrated in Figure 2.

The half-maximal inhibitory concentration (IC_50_) of each compound was obtained by nonlinear regression, and the results are shown in Table 1. The IC_50_ of the tested compounds ranged from 3.96 to 40.98 µM for promastigote forms and from 15.93 to >50 µM for amastigote forms. Amphotericin B, a reference drug for its antileishmanial activity, was also evaluated under the experimental conditions, obtaining an IC_50_ of 9.40 µM for promastigotes and 0.97 µM for intracellular amastigote forms.

The highest activity against promastigote forms of *L. amazonensis* was observed for compound **MRK-106**, which contains a 4,5-dihydrooxazole ring in its structure and showed an IC_50_ of 3.96 µM. Compound **MRK-108**, a selenocyanate linked to a coumarin ring, had an IC_50_ of 4.23 µM, the second-highest potency observed against the parasite. Following the decreasing order of antileishmania activity observed, the following compounds showed similar activity between 10 and 20 µM: **MRK-105**, a phenol selenocyanate, showed an IC_50_ of 12.17 µM; **MRK-102**, a beta-naphthol, showed an IC_50_ of 15.15 µM; compound **MRK-103**, which has a 1,3,4-oxadiazole ring, showed an IC_50_ of 15.48 µM; **MRK-104**, which has an indolic nucleus, showed an IC_50_ of 16.17 µM; and compound **MRK-111**, an imidazothiazole, showed an IC_50_ of 17.55 µM. The least active compounds had IC_50_s above 20 µM: **MRK-107**, an imidazopyridine, had an IC_50_ of 27.37 µM; **MRK-101**, a chromone, had an IC_50_ of 30.46 µM; and **MRK-113**, also an imidazopyridine, had an IC_50_ of 40.98 µM.

It is interesting to note that the activity of the compounds against promastigote forms did not necessarily reflect the results of the anti-amastigote intracellular activity experiments: the compounds **MRK-106** and **MRK-108** were not active in macrophages infected with *L. amazonensis*, with IC_50_ values estimated at >50 µM, the highest concentration tested. On the other hand, the compounds **MRK-107** and **MRK-113**, which were poorly active against promastigote forms, showed the best results against intracellular amastigote forms: 18.31 and 15.93 µM, respectively. As most of the tested compounds did not show activity against intracellular amastigotes, their SIs were considered indeterminate (indicated as *ind* in Table 1).

### 3.2. Cytotoxicity and SI

**Figure 3:**
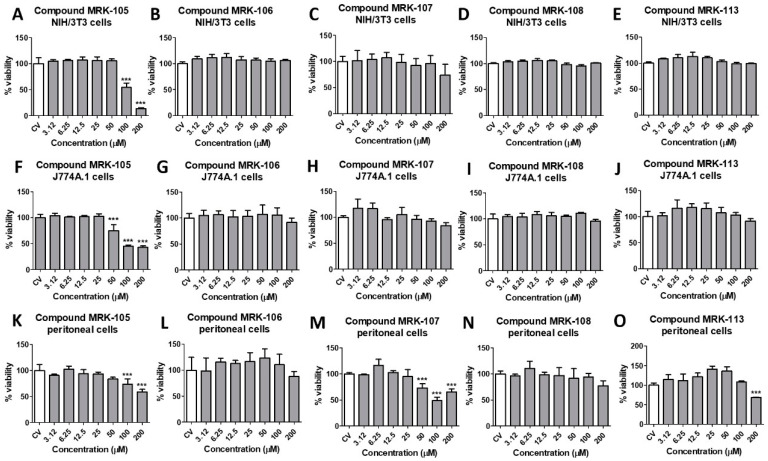
Cytotoxicity of synthesized selenium compounds **MRK-105**, **MRK-106**, **MRK-107**, **MRK-108**, and **MRK-113** against NIH/3T3 fibroblasts (**A**–**E**, upper graphs), J774A.1 macrophages (**F**–**J**, middle graphs) and murine peritoneal macrophages (**K**–**O**, lower graphs). *** mean *p* < 0.001 (ANOVA and Tukey´s post-test).

The SI is defined as the ratio of the CC_50_ obtained for the host cells to the IC_50_ obtained against the parasite cells and helps identify the compounds that exhibit a high degree of specificity in targeting the pathogen of interest while sparing the host cells [42,43]. To determine the SI of the selenium compounds, their cytotoxicity on murine peritoneal macrophages, NIH/3T3, and J774A.1 cells was also evaluated at concentrations ranging from 200 to 3.12 µM. After 48 h, their viability was assessed using the resazurin colorimetric methodology, where cells incubated only with culture medium were considered 100% viable. The representative results obtained for five of the ten compounds tested are illustrated in Figure 3. The CC_50_ was obtained by nonlinear regression, and the results for the CC_50_ for peritoneal macrophages are also shown in Table 1. The CC50 and SI results for NIH/3T3 and J774A.1 cells for each compound are shown in the Supplemental Materials.

All the tested selenium compounds showed a CC_50_ above 200 µM in peritoneal macrophages. Some compounds showed higher cytotoxicity in other cell lines: compound **MRK-105**, whose CC_50_ was 87.85 µM in NIH/3T3 cells and 60.16 µM in J774A.1 cells, and compound 104 with the CC_50_ of 126.7 µM in J774A.1 cells. Thus, the most active compounds against *L. amazonensis* promastigotes were those with a higher SI, compounds **MRK-106** and **MRK-108**, with SIs above 50.53 and 47.31, respectively. However, considering the cytotoxicity in peritoneal macrophages and the activity against intracellular amastigotes of *L. amazonensis*, the most active compounds were **MRK-113** and **MRK-107**, with SI values of 12.55 and 10.92, respectively.

## 4. Discussion

Leishmaniasis, a neglected tropical disease, continues to pose a significant challenge for drug discovery and development. Current antileishmanial drugs have issues such as high toxicity, resistance development, and the necessity for hospitalization, resulting in poor patient adhesion [3,4]. While notable strides have been made through combination therapy approaches, which have reduced treatment duration and cost, a critical gap persists—the urgent need for new active drugs. In the search for novel drugs, emerging evidence highlights a link between selenium and parasites, notably trypanosomatids. Certain parasites have been found to both express selenoproteins and metabolize selenium. This underscores the potential significance of selenium as a promising element for the development of new agents against leishmaniasis. Here, we demonstrated that using sustainable routes, synthesized (hetero)aryl hybrid selenium compounds displayed anti-promastigote activity in vitro and a promising SI, with **MRK-106**, **MRK-107**, **MRK-108**, and **MRK-113** being the most potent and selective for antileishmanial activity.

**MRK-106** contains a 4,5-dihydrooxazole ring (oxazoline), with substituents containing benzene rings in positions 2 and 5, showing an IC_50_ of 3.96 µM for promastigotes and an SI of 50.53. Secondary metabolites featuring oxazole, oxazoline, and isoxazoline ring structures exhibit a broad distribution across marine and terrestrial organisms [44,45]. Among various heterocyclic compounds, isoxazoles and their analogs hold great significance due to their wide-ranging biological activities. This makes them pivotal structures in medicinal chemistry. Isoxazole derivatives with structural variations exhibit diverse medicinal properties, contributing significantly to the development of novel, highly effective, and less toxic bioactive drugs. These compounds are notable for their diverse and substantial biological activities, encompassing antitumor, antibacterial, antiviral, anti-malarial, and immunosuppressive properties, including antileishmanial activity [44]. Moraski and colleagues synthesized multiple compounds with oxazoline and oxazole motifs and tested for their inhibitory potential against *Mycobacterium tuberculosis*, showing promising results [45,46]. More recently, a series of compounds containing β-carboline-oxazoline were tested against promastigote and amastigote forms of *L. amazonensis*, and some of them were found to be active against the parasite [47]. Among them, compounds 8d and 8i were considered the most potent against promastigote forms, showing an IC_50_ of 14.7 and 23 µM and an SI of 6.6 and 1.3, respectively. Our compound **MRK-106** also has an oxazoline ring in its structure, together with a Se atom and two benzene rings, which resulted in a lower IC_50_ and a higher SI compared to the β-carboline-oxazoline molecules. Another compound containing an azol is **MRK-103**, which has a 1,3,4-oxadiazole ring. Despite several examples of using different azol moieties in active antileishmanial compounds [44], **MRK-105** showed an IC_50_ of 15.48 µM and an SI of 12.92, more than three times less selective than **MRK-108**. It is also interesting to note that, although a higher IC_50_ was obtained than for amphotericin B under the experimental conditions used, the SI for **MRK-106** (50.53) was more than five times higher than that obtained for this drug (9.40) for promastigote forms. Amphotericin B is an important second-choice drug in the treatment of leishmaniasis, but it is also known for its toxic side effects and low therapeutic index [3,4]. Thus, the SI obtained for **MRK-106** is also an important datum for future studies of antileishmanial activity.

Compound **MRK-108**, a selenocyanate linked to a coumarin ring, showed an IC_50_ of 4.23 µM and an SI of 47.31. The potential for functionalization and distinctive attributes renders coumarin a privileged scaffold in the field of medicinal chemistry [48]. While coumarins are predominantly found as secondary metabolites in plants, bacteria, and fungi, numerous synthetic methods have been documented for their production. This bicyclic heterocycle, composed of a benzene ring fused with a pyrone ring, exhibits the capability to engage with diverse biological targets [48]. The pyrone ring facilitates hydrogen bonding with multiple amino acid residues, while the aromatic segment can establish hydrophobic interactions. Consequently, this versatility results in a wide array of biological properties, encompassing antioxidant, anticoagulant, anticancer, antiviral, antitrypanosomal, anticholinesterase, and antileishmanial activities. Coumarin derivatives were identified as promising structures in the search for new antileishmania agents in a recent review [49]. Also, the authors pointed out that the presence of electron-withdrawing groups increases the antileishmanial effect. In addition, selenocyanates displayed potency against *Leishmania infantum* promastigotes [24]. In the case of compound **MRK-108**, the selenium atom is linked to the nitrile (strongly electron-withdrawing group) [50,51] and on the other side to methyl-2*H*-pyran-2-one of coumarin, and this may contribute to the activity observed for **MRK-108**. The authors also highlight the work of Huang and colleagues with quinoline derivatives containing selenium as promising antileishmanial candidates [49,51,52]. The SI obtained for **MRK-108** (47.31) was also almost three times higher than that of amphotericin B, which could be a promising characteristic for the therapeutic use of this molecule or a series of derivative compounds for further structure–activity relationship studies.

**MRK-107** and **MRK-113** showed low activity against *L. amazonensis* promastigotes (27.37 and 40.98 µM, respectively), and it is interesting to observe that both are imidazopyridine compounds, with a phenyl-selenyl substituent in carbon 3. However, the presence of a methoxy group for **MRK-107** enhanced its activity almost 1.5x compared to **MRK-113**, without methoxy, an electron-donating group. This should contribute to the antileishmanial activity and, like other compounds studied here, further structure-related activity should be carried out to address the ligand and/or the essential radicals responsible for the observed activity. Despite the results obtained for promastigote cells, both **MRK-107** and **MRK-113** were the most active compounds against intracellular amastigotes, with an IC_50_ of 18.31 and 15.93 µM and an SI of 12.55 and 10.92, respectively. The imidazopyridine scaffold has gained significant importance for designing synthetic analogs targeting a range of therapeutic disorders, including cancer, diabetes, infections, inflammation, and central nervous system (CNS) conditions. This heterocyclic system serves as a crucial pharmacophore motif, expanding medicinal chemistry tools. Additionally, imidazopyridines are used in combating helminthic, coccidial, and fungal infections, illustrating their multifaceted role in drug development [53]. Recently, imidazopyridine derivatives have been reported to have the potential for antitrypanosomiases drug discovery. Fersing et al. designed and synthesized novel 3-nitroimidazo[1,2-a]-pyridine derivatives. By introducing a heteroatom bridge between the aryl group and the imidazopyridine, they obtained the desired derivatives from 8-bromo-6-chloro-3-nitro-2-(phenylsulfonylmethyl)imidazo[1,2-a]pyridine. These compounds were tested in vitro against *Leishmania donovani* and *L. infantum* strains alongside reference drugs like pentamidine, fexinidazole, miltefosine, and amphotericin B [54]. Such structural features can serve as a basis for the design and synthesis of a new series of imidazopyridines containing selenium.

Various chemical structures containing selenium have been investigated against *Leishmania* parasites, including diselenide, selenourea, methylseleno, and selenocyanate components [22]. Both **MRK-105** and **MRK-108** contain selenocyanate radicals, but their structures are quite different: **MRK-105** is a phenol selenocyanate, while **MRK-108** is a selenocyanate linked to a coumarin, as described above. Also, **MRK-8** showed higher activity against *L. amazonensis* promastigotes (4.23 µM) than **MRK-105** (12.17 µM), despite its poor activity against intracellular amastigotes. Regarding the SI, **MRK-105** was the only compound whose cytotoxicity in NIH/3T3 fibroblasts was lower than 200 µM (87.85 µM), resulting in a low SI of 7.22. Also, **MRK-104** and **MRK-105** showed higher cytotoxicity in J774A.1 cells: 126.7 and 60.16 µM, respectively. **MRK-8**, by its turn, exhibited an SI of 47.31. These results highlight the promising structure of selenocyanates associated with the coumarin rings in the search for novel antileishmanial drugs.

Based on the recommendations of the WHO Special Program for Tropical Disease Research (TDR), some authors classify the compounds tested for *L. donovani* or *L. infantum* as active for an IC_50_ in amastigotes in macrophages of 1–2 µg/mL, moderately active for an IC_50_ between 1.0 and 6.0 µM, and inactive for an IC_50_ > 6.0 µM, with a desirable SI > 10 or even > 20 [42,55,56]. This is an important step regarding antileishmanial potential, as intracellular amastigotes are the parasite forms found in mammalian hosts. However, testing new compounds against promastigote forms of *Leishmania* is still an important step in the search for their biological activity, as recently reviewed, considering natural and synthetic compounds with antileishmanial activity [57,58]. So, further evaluation of these selenium compounds in other *Leishmania* species or even modifications of their structures should be performed to outline their promising antileishmanial activity.

This study used established in vitro models of immortalized NIH/3T3 and J774A.1 cell lines for cytotoxicity and BALB/c mouse peritoneal macrophages for both cytotoxicity and intracellular anti-amastigote activity assays. These models provide valuable first insights into compound activity, mimic parasite–host interactions, and facilitate initial compound screening, despite their limitations [42]. Future studies may include human cell models, in vivo testing in infected BALB/c mice, and comprehensive post-administration safety and efficacy evaluations.

## 5. Conclusions

In conclusion, a series of selenium-substituted (hetero)aryl hybrids was screened against *L. amazonensis* promastigotes and intracellular amastigotes. These hybrid compounds included selenium-substituted indole, coumarin, chromone, oxadiazole, imidazo[1,2-*a*]pyridine, Imidazo[2,1-*b*]thiazole, and oxazole, among others. The biological evaluation indicates that these hybrids are promising structures against this parasite. Furthermore, their cytotoxicity was also assessed in murine peritoneal macrophages, NIH/3T3, and J774A.1 cells with good selectivity. Compounds containing oxazoline rings, coumarin derivatives, and imidazopyridine rings showed the best parameters for evaluating antileishmanial activity and selectivity. These compounds may serve as a basis for the synthesis of structural derivatives and thus develop more detailed structure–activity studies. The data in this study represent an important step toward the search for new antileishmanial drugs to be explored further.

## Figures and Tables

**Table 1:** Antileishmanial activity of selenium compounds.

Compound	CC_50_ (µM) Peritoneal Macrophages	IC_50_ (µM) *L. amazonensis* Promastigotes	SI ^a^	IC_50_ (µM) *L. amazonensis* Amastigotes	SI ^b^
**101**	>200	30.46 ± 1.17	6.57	>50	ind
**102**	>200	15.15 ± 1.12	13.20	>50	ind
**103**	>200	15.48 ± 1.13	12.92	>50	ind
**104**	>200	16.17 ± 1.19	12.37	>50	ind
**105**	>200	12.17 ± 1.12	16.43	>50	ind
**106**	>200	3.96 ± 1.09	50.53	>50	ind
**107**	>200	27.37 ± 1.24	7.31	18.31 ± 1.50	10.92
**108**	>200	4.23 ± 2.00	47.31	>50	ind
**111**	>200	17.55 ± 1.18	11.40	>50	ind
**113**	>200	40.98 ± 1.31	4.88	15.93 ± 1.69	12.55
ANFB	25.15 ± 0.82	9.40 ± 1.13	2.67	0.97 ± 1.14	25.92

The antileishmanial activity of selenium compounds was evaluated against promastigote and amastigote forms of *L. amazonensis* after 48 and 24 h, respectively. The cytotoxicity was performed in BALB/c peritoneal macrophages. The selectivity index (SI) was calculated as the CC_50_/IC_50_ values for promastigote and amastigote forms, respectively. Abbreviations: CC_50_ = half-maximal cytotoxic concentration; IC_50_ = half-maximal inhibitory concentration; SD = standard deviation; SI = selectivity index. ^a^ SI, CC_50_ on murine peritoneal macrophages/IC_50_ on promastigotes. ^b^ SI, IC_50_ on murine peritoneal macrophages/IC_50_ on amastigotes. ANFB = amphotericin B used as a reference drug for *L. amazonensis*. Ind = indeterminate results.

## Data Availability

Data are contained within the article and Supplementary Materials.

## Supplementary Materials

The following supporting information can be downloaded at: https://www.mdpi.com/article/10.3390/biomedicines12010213/s1, CC50 and SI results for NIH/3T3 and J774A.1 cells for each compound are shown in Supplemental Material (Tables S1 and S2).
