# Supplementary material for: Selenium-Containing (Hetero)Aryl Hybrids as Potential Antileishmanial Drug Candidates: In Vitro Screening against L. amazonensis
Source: Biomedicines. 2024 Jan 17;12(1):213. doi: 10.3390/biomedicines12010213 (PMC10812941; doi:10.3390/biomedicines12010213)
Supplement: Supplementary file 1 [file biomedicines-12-00213-s001.zip › biomedicines-2670671-supplementary.pdf]

TABLE S1. Cytotoxicity and selectivity index (SI) of selenium-compounds against different host cell lines and promastigote forms of *Leishmania amazonensis*.

Compound	IC50 *L. amazonensis* (μM)	CC50 NIH/3T3 (μM)	Selectivity Index (SI)	CC50 J774 (μM)	Selectivity Index (SI)	CC50 peritoneal cells (μM)	Selectivity Index (SI)
**101**	30.46±1.17	> 200	6.57	> 200	6.57	> 200	6.57
**102**	15.15±1.12	> 200	13.20	> 200	13.20	> 200	13.20
**103**	15.48±1.13	> 200	12.92	> 200	12.92	> 200	12.92
**104**	16.17±1.19	> 200	12.37	126.7±1.04	7.84	> 200	12.37
**105**	12.17±1.12	87.85±1.09	7.22	60.16±1.04	4.94	> 200	16.43
**106**	3.96±1.09	> 200	50.53	> 200	50.53	> 200	50.53
**107**	27.37±1.24	> 200	7.31	> 200	7.31	> 200	7.31
**108**	4.23±2.00	> 200	47.31	> 200	47.31	> 200	47.31
**111**	17.55±1.18	> 200	11.40	> 200	11.40	> 200	11.40
**113**	40.98±1.31	> 200	4.88	> 200	4.88	> 200	4.88
ANFB	9.407±1.13	19.26±1.01	2.05	27.34±0.92	2.91	25.15±0.82	2.67

The antileishmanial activity of selenium-compounds was evaluated against promastigote forms of *L. amazonensis* after 48 hours. The cytotoxicity was performed in NIH/3T3 cells, J774A.1 cells and peritoneal macrophages. Selectivity index (SI) was calculated as CC50/IC50 values for Abbreviations: CC50 = half-maximal cytotoxic concentration; IC50 = half-maximal inhibitory concentration; SD = standard deviation; SI = selectivity index. ANFB = amphotericin B used as reference drug for *L. amazonensis*.

TABLE S2. Cytotoxicity and selectivity index (SI) of selenium-compounds against different host cell lines and intracellular amastigotes forms of *Leishmania amazonensis*.

Compound	IC50 *L. amazonensis* (μM)	CC50 NIH/3T3 (μM)	Selectivity Index (SI)	CC50 J774 (μM)	Selectivity Index (SI)	CC50 peritoneal cells (μM)	Selectivity Index (SI)
**101**	> 50	> 200	ind	> 200	ind	> 200	ind
**102**	> 50	> 200	ind	> 200	ind	> 200	ind
**103**	> 50	> 200	ind	> 200	ind	> 200	ind
**104**	> 50	> 200	ind	126.7±1.04	ind	> 200	ind
**105**	> 50	87.85±1.09	ind	60.16±1.04	ind	> 200	ind
**106**	> 50	> 200	ind	> 200	ind	> 200	ind
**107**	18.31±1.50	> 200	10.92	> 200	10.92	> 200	10.92
**108**	> 50	> 200	ind	> 200	ind	> 200	ind
**111**	> 50	> 200	ind	> 200	ind	> 200	ind
**113**	15.93±1.69	> 200	12.55	> 200	12.55	> 200	12.55
ANFB	0.97±1.14	19.26±1.01	25.92	27.34±0.92	28.19	25.15±0.82	25.93

The antileishmanial activity of selenium-compounds was evaluated against intracellular amastigotes forms of *L. amazonensis* after 24 hours. The cytotoxicity was performed in NIH/3T3 cells, J774A.1 cells and peritoneal macrophages. Selectivity index (SI) was calculated as CC50/IC50 values for Abbreviations: CC50 = half-maximal cytotoxic concentration; IC50 = half-maximal inhibitory concentration; SD = standard deviation; SI = selectivity index. ANFB = amphotericin B used as reference drug for *L. amazonensis*. Ind = means indetermined results.
